# PREVALENCE OF HEPATIC ARTERIAL VARIATIONS WITH IMPLICATIONS IN PANCREATODUODENECTOMY

**DOI:** 10.1590/0102-672020190001e1455

**Published:** 2019-10-21

**Authors:** Silvio Marcio Pegoraro BALZAN, Vinicius Grando GAVA, Sabrina PEDROTTI, Marcelo Arbo MAGALHÃES, Alex SCHWENGBER, Marcelo Luiz DOTTO, Carmela Reckziegel KREBS

**Affiliations:** 1 Department of Biology and Pharmacy, Medicine Course, University of Santa Cruz do Sul, Santa Cruz do Sul;; 2 Institute of Oncology Saint Gallen, Hepatobiliary-pancreatic Surgery, Santa Cruz do Sul;; 3 Hospital Moinhos de Vento, Oncology Center Lydia Wong Ling, Porto Alegre;; 4 Radson, Ana Nery Hospital, Santa Cruz do Sul, RS, Brazil

**Keywords:** Pancreaticoduodenectomy, Hepatic artery, Pancreatic neoplasms, Pancreaticoduodenectomia, Artéria hepática, Neoplasias pancreáticas

## Abstract

**Background:**

Pancreaticoduodenectomy is the usual surgical option for curative treatment of periampullary cancer and carries a significant mortality. Arterial anomalies of the celiac axis are not uncommon and might lead to iatrogenic lesions or requiring arterial resection/reconstruction in a pancreatoduodenectomy.

**Aim:**

Determine the prevalence of arterial variations having implications in pancreatoduodenectomy.

**Methods:**

Celiac trunk and hepatic arterial system anatomy was retrospectively evaluated in 200 abdominal enhanced computed tomography studies.

**Results:**

Normal anatomy of hepatic arterial system was found in 87% of cases. An anomalous right hepatic artery was identified in 13% of cases. In 12 cases there was a substitute right hepatic artery arising from superior mesenteric artery and in two cases an accessory right hepatic artery with similar origin. A hepatomesenteric trunk was identified in seven cases and in five there was a right hepatic artery directly from the celiac trunk. All cases of anomalous right hepatic artery had a route was behind the pancreatic head and then, posteriorly and laterally, to the main portal vein before reaching the liver.

**Conclusions:**

Hepatic artery variations, such as anomalous right hepatic artery crossing posterior to the portal vein, are frequently seen (13%). These patients, when undergoing pancreatoduodenectomy, may require a change in the surgical approach to achieve an adequate resection. Preoperative imaging can clearly identify such variations and help to achieve a safer pancreatic head dissection with proper surgical planning.

## RESUMO

**Racional:**

Pancreatoduodenectomia consiste no procedimento cirúrgico usual para tratamento curativo de neoplasias periampulares e apresenta mortalidade significativa. Variações arteriais do tronco celíaco não são incomuns e podem favorecer lesões iatrogênicas ou exigirem realização de ressecção/reconstrução arterial durante pancreatoduodenectomia.

**Objetivo:**

Determinar a prevalência de variações arteriais que apresentam implicações durante pancreatoduodenectomia.

**Métodos:**

A anatomia do tronco celíaco e sistema arterial hepático foi investigada retrospectivamente em 200 exames tomográficos contrastados do abdome.

**Resultados:**

Anatomia normal do sistema arterial hepático foi observada em 87% dos casos. Presença de uma artéria hepática direita anômala foi identificada em 13%. Em 12 casos houve uma artéria hepática direita substituta originária da artéria mesentérica superior, em dois uma artéria hepática direita acessória com origem similar. Tronco hepaticomesentérico foi identificado em sete casos e em cinco houve uma artéria hepática direita originária diretamente do tronco celíaco. Em todos casos de artéria hepática direita anômala seu curso foi por trás da cabeça do pâncreas e com trajeto passando posteriormente ao tronco da veia porta e após percorrendo sua face lateral direita antes de alcançar o fígado.

**Conclusões:**

Variações arteriais hepáticas, como artéria hepática direita anômala com trajeto posterior à veia porta, são frequentes (13%). Nestes pacientes, quando submetidos à pancreatoduodenectomia, pode ser necessária alteração na abordagem cirúrgica para ressecção adequada. Exames de imagem pré-operatórios podem claramente identificar estas variações e auxiliar na realização de dissecção segura da cabeça do pâncreas com adequado planejamento cirúrgico.

## INTRODUCTION

Surgical resection remains the only potentially curative treatment for cancer of the head of the pancreas, and pancreatoduodenectomy (PD) is the standard surgical option^8^ ^,^ ^10^. Despite technical advances, PD remains a challenging operation with surgical mortality rates ranging from 1-6% even at experienced centers^17^ ^,^ ^19^ ^,217,^ ^10^. Major vessels involvement around the pancreas can make the surgery even more challenging^4^ ^,^ ^6^ ^,^ ^23^ especially if arterial resection and reconstruction are necessary^1^ ^,^ ^13^ ^,^ ^14^ ^,^ ^16^. Arterial anomalies of the celiac axis, mainly of hepatic arteries, are not uncommon. These variations can result in arterial involvement of the anomalous artery by the tumor and increase the risk of vascular injury. Thus, awareness of patient vascular anatomy is important to avoid iatrogenic injury while performing a safe pancreatic head resection. 

The purpose of this study was to determine the prevalence of arterial variations that can lead to iatrogenic injury or require resection/reconstruction during a PD.

## METHOD

### Patients

Two hundred consecutive patients were submitted to contrast enhanced abdominal computed tomography (CT) in a tertiary radiology unit. CTs were retrospectively analyzed. Patients with a previous history of major upper abdominal surgery, a large abdominal mass that distorted the celiac or its branches, and those younger than 18 years old were excluded. This study was submitted and approved by the institutional ethics committee (number 2.755.349) and registered at *Plataforma Brasil*.

### Image technique and interpretation

The CT images were obtained with the use of a spiral 16-detector row scanner (LightSpeed Ultra Scanner, GE Medical Systems). Section thicknesses of 3.0 mm or less was used. Dynamic images were obtained after endovenous injection of iopromide through an 18-gauge plastic catheter placed in an antecubital vein. The hepatic arterial phase scanning delays 9-15 seconds after descending aorta enhancement of 100 Housfied Units (HU). Total contrast medium volume used ranged from 1-1.5 ml per kg of body weight. Hepatic arterial, portal venous, and equilibrium phases were routinely achieved. Dicom images were analyzed by a radiologist and a hepatobiliarypancreatic surgeon using a free open source medical image viewer (Horos 3.3.1 for MacOS, Horos project, 2018).

### Arterial anomalies

Standard arterial anatomy was considered as: the common hepatic artery originating from celiac trunk and being called proper hepatic artery after giving the gastroduodenal artery. Right and left hepatic arteries originating from proper hepatic artery. The presence of a right hepatic artery (RHA) (replaced or accessory) or a common hepatic artery originating from the superior mesenteric artery are known arterial anomalies that require special caution and/or technical modifications during PD. These variations were searched in each tomographic study and recorded in schematic figures. The Hiatt classification was adopted to evaluate arterial anatomical variations^9^.

## RESULTS

A total of 214 patients were initially screened from June to August 2018 in a tertiary radiology unit. There were 14 exclusions due to large abdominal tumors (four cases of abdominal neoplasms and one of polycystic hepatic disease) distorting the celiac trunk and 10 exclusions due to major upper abdominal surgery (five pancreatic resection and other five major hepatic resection).The distribution of men and women was similar (93 and 107, respectively).

**FIGURE 1:**
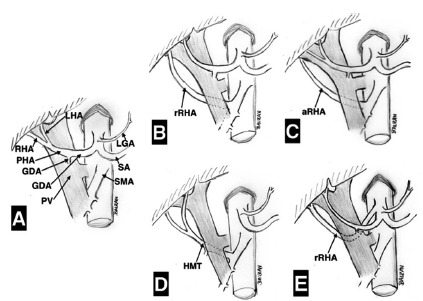
Diagrammatic representation of hepatic arterial system variations with implications in pancreatoduodenectomy observed in 200 reviewed cases. Variations of left hepatic artery (n=7) are not shown: A) standard arterial configuration; B) replaced right hepatic artery from superior mesenteric artery (prevalence 6%); C) accessory right hepatic artery from superior mesenteric artery (prevalence 1%); D) right hepatic artery from hepatomesenteric trunk (prevalence 3.5%); and E) replaced right hepatic artery directly from celiac trunk with retroportal course (prevalence 2.5%).

**FIGURE 2:**
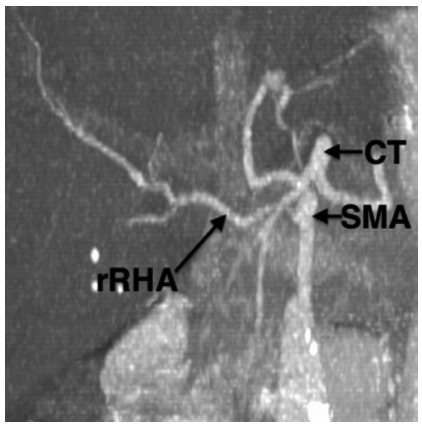
Replaced right hepatic artery (rRHA) arising from superior mesenteric artery (SMA). CL=celiac trunk.

A normal RHA originating from the main trunk of the proper hepatic artery (Figure 1A) was found in 174 cases (87%). An aberrant RHA was identified in 26 (13%). Among these 26 cases of aberrant RHA, 12 (6%) were a replaced RHA arising from SMA (Figure 1B and Figure 2), two (1%) were an accessory RHA arising from SMA (Figure 1C), seven (3.5%) the origin of the hepatic artery was a hepatomesenteric trunk (Figure 1D), and five (2.5%) the RHA was replaced with its origin directly from celiac trunk (Figure 1E). All aberrant RHA (13%) had a route behind the head of the pancreas and then, posteriorly and laterally, to the main portal vein before reaching the liver.

LHA=left hepatic artery; RHA=right hepatic artery; PHA=proper hepatic artery; GDA=gastroduodenal artery; PV=portal vein; LGA=left gastric artery; SA=splenic artery; SMA=superior mesenteric artery; rRHA=replaced right hepatic artery; aRHA=accessory right hepatic artery; HMT=hepatomesenteric trunk

Additionally, eight cases (4%) of aberrant left hepatic artery were identified. In seven cases the left hepatic artery (replaced in four cases and accessory in three) arose from the left gastric artery. In one case a replaced left hepatic artery came directly from aorta. The course of these aberrant left hepatic arteries was far from head of the pancreas. In one of the reported cases there was an association of accessory left hepatic artery and replaced right hepatic artery. Also, there was one case celiac trunk and superior mesenteric artery arising from a common trunk from aorta.

## DISCUSSION

Following normal development, common hepatic artery arises from celiac trunk and it continues as the proper hepatic artery after giving origin to the gastroduodenal artery. Right and left hepatic arteries usually are branches of the proper hepatic artery. However, numerous anatomical arterial variations can result from anomalies of embryologic elements^22^. In fact, a “normal” or regular arterial pattern of arterial hepatic branches is reported with a frequency of 62.5% to 90.5% of cases^15^ ^,^ ^22^ ^,^ ^26^. Some anatomical variations of hepatic arterial system have crucial importance in pancreatic head resections, such as PD^2^ ^,^ ^11^ ^,^ ^18^.

Since the initial PD description by Whipple et al.^25^ in 1935, this complex procedure evolved and underwent several changes^3^. However, arterial anatomical variations remain a challenge. Ligation of a hepatic artery may result in hepatic necrosis, liver abscesses, ischemic biliary injury, and/or an anastomotic fistula. These are potential life-threatening complications^13^. 

A standard PD and its variations comprise at least dissection of common and proper hepatic arteries and gastroduodenal artery. This one is usually ligated and sectioned in its origin. Also, lymphadenectomy of the common hepatic artery and celiac trunk is frequently indicated in PD for cancer. Additionally, pancreatic arterial branches from superior mesenteric artery (such as inferior pancreatoduodenal arteries) are ligated during retroportal pancreatic lamina resection^20^.

Anomalous right hepatic artery originating from the superior mesenteric artery might show different relations with the pancreas and the portal vein. They are often in contact with the posterior aspect of the head of the pancreas and go lateral and posterior to the portal vein. Anomalous common hepatic artery arising from the SMA (known as the hepatomesenteric trunk) might have a similar course. An important finding of the present study is that in all cases of anomalous right hepatic artery, including those that arouse directly from SMA and also those from a hepatic-mesenteric trunk, run posterior to the pancreatic head and portal vein, and laterally to the portal vein before reaching the liver. These anomalous vessels might be accidentally damaged during PD and/or can be involved by tumors of the pancreatic head and cause intra-operative or postoperative bleeding.^13^ ^,^ ^14^

**FIGURE 3:**
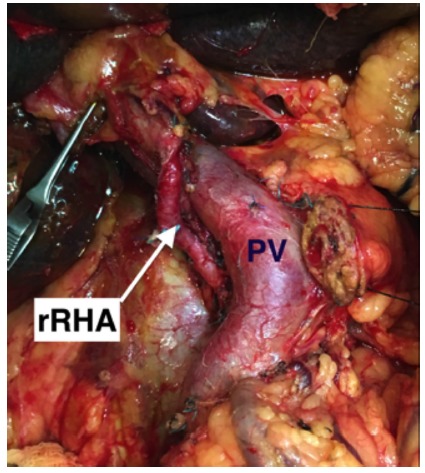
Operative view showing dissection of a replaced right hepatic artery (rRHA) during a pancreatoduodenectomy: note the course of rRHA posterior to the portal vein (PV) and then lateral to the PV.

**FIGURE 4:**
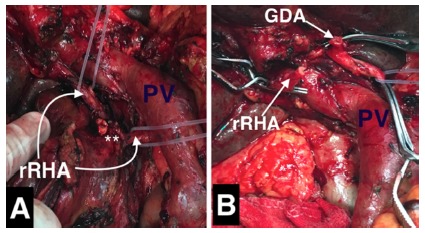
Operative view during a pancreatoduodenectomy: A) note the presence of a replaced right hepatic artery (rRHA) from superior mesenteric artery, crossing posterior to the portal vein (PV) and involved by a pancreatic tumor (asterisks); B) segmental resection of the involved rRHA with distal segment prepared to be anastomosed with the stump of the gastroduodenal artery (GDA)

Identification of arterial variations allows proper planning and appropriate operative management in case of vascular encasement or arterial injury. Simple ligation and section should be avoided (except in cases of accessory vessels) due to the risk of hepatic necrosis and liver abscess. Despite of being technically demanding, dissection of the vessels far from the pancreas is usually possible without compromising the radicality of resection (Figure 3) and should be attempted^2^. Resection and reconstruction may be required in certain arterial variations (such as in case of intra-pancreatic course) or tumor encasement (Figure 4)^2^ ^,^ ^18^. It has been demonstrated that the presence of a anomalous RHA in patients with pancreatic adenocarcinoma does not affect resectability. It is suggested that in patients with resectable pancreatic tumors the presence of this variation does not increase R1 rates and is not associated with worse postoperative outcomes or overall survival^5^ ^,^ ^12^ ^,^ ^24^. In most of cases of PD with an aberrant RHA it was possible to preserve this artery; resection and reconstruction was restricted to arterial encasement. For a while, it is not clear if arterial involvement of an aberrant RHA by a pancreatic tumor has the same clinical impact than involvement of other arteries (such as celiac trunk or common hepatic artery). Thus, resection of a tumor involved aberrant right hepatic artery might represent an acceptable option.

It was found an anomalous right hepatic artery (either an accessory or substitute RHA from SMA or a RHA from a hepatic-mesenteric trunk) in 26 cases (13%), all of them with a course just posterior to the portal vein and in contact with the head of the pancreas. Additionally, were found two cases of a RHA directly from the celiac trunk, also with a retroportal course. These findings are accordance with the literature, with a 13-26% rate of anomalous RHA reported^11^ ^,^ ^26^.

The definition of the arterial vascular anatomy preoperatively or early during PD is crucial. It can avoid bleeding and postoperative complications due to arterial injury. Associated with the current imaging techniques, dissection of the superior mesenteric artery as an initial step of PD can be used very selectively.

## CONCLUSION

Hepatic artery variations, such as anomalous right hepatic artery crossing posterior to the portal vein, are frequently seen. These patients, when undergoing pancreatoduodenectomy, may require a change in the surgical approach to achieve an adequate resection. Preoperative imaging can clearly identify such variations and help to achieve a safer pancreatic head dissection with proper surgical planning.
